# Kinase-independent function of RIP1, critical for mature T-cell survival and proliferation

**DOI:** 10.1038/cddis.2016.307

**Published:** 2016-09-29

**Authors:** John P Dowling, Yubo Cai, John Bertin, Peter J Gough, Jianke Zhang

**Affiliations:** 1 Department of Microbiology and Immunology, Sidney Kimmel Cancer Center, Thomas Jefferson University, 233S., 10th Street, Philadelphia, PA 19107, USA; 2 Pattern Recognition Receptor Discovery Performance Unit, Immuno-inflammation Therapeutic Area, GlaxoSmithKline, Collegeville, PA 19426, USA

## Abstract

The death receptor, Fas, triggers apoptotic death and is essential for maintaining homeostasis in the peripheral lymphoid organs. RIP1 was originally cloned when searching for Fas-binding proteins and was later shown to associate also with the signaling complex of TNFR1. Although Fas exclusively induces apoptosis, TNFR1 primarily activates the pro-survival/pro-inflammatory NF-*κ*B pathway. Mutations in Fas lead to lymphoproliferative (*lpr*) diseases, and deletion of TNFR1 results in defective innate immune responses. However, the function of RIP1 in the adult lymphoid system has not been well understood, primarily owing to perinatal lethality in mice lacking the entire RIP1 protein in germ cells. This current study investigated the requirement for RIP1 in the T lineage using viable RIP1 mutant mice containing a conditional and kinase-dead RIP1 allele. Disabling the kinase activity of RIP1 had no obvious impact on the T-cell compartment. However, T-cell-specific deletion of RIP1 led to a severe T-lymphopenic condition, owing to a dramatically reduced mature T-cell pool in the periphery. Interestingly, the immature T-cell compartment in the thymus appeared intact. Further analysis showed that mature RIP1^−/−^ T cells were severely defective in antigen receptor-induced proliferative responses. Moreover, the RIP1^−/−^ T cells displayed greatly increased death and contained elevated caspase activities, an indication of apoptosis. In total, these results revealed a novel, kinase-independent function of RIP1, which is essential for not only promoting TCR-induced proliferative responses but also in blocking apoptosis in mature T cells.

The pro-survival signaling pathways provide protection against cell death responses at various stages during T lymphopoiesis as well as maintenance of the mature population.^1, 2^ Apoptosis is a major programmed cell death pathway, which can be induced through either intrinsic or extrinsic signals.^3^ Under normal circumstances, the pro-survival and apoptosis signaling pathways are tightly regulated, which ensures generation of diverse T-cell repertoires, while avoiding autoimmunity. For instance, the Bcl-2 and Bcl-X_L_ genes, which inhibit the intrinsic apoptotic pathway, are essential for both T-cell development and peripheral maintenance.^4, 5^ However, lack of cell death, as in the case of inactivation of Bim, a pro-apoptotic protein of the Bcl-2 family, results in lymphoproliferative and autoimmune diseases.^6^ The extrinsic pathway of apoptosis is triggered through cell receptors, including Fas/Apo-1 and tumor necrosis factor receptor 1 (TNFR1). Whereas Fas is a professional death receptor, TNFR1 primarily signals the pro-survival pathway by activating NF-*κ*B, which also promotes inflammation.^7, 8^

Receptor-interacting protein (RIP or RIP1) was originally cloned as a potential Fas-interacting protein.^9^ However, later studies found that lack of RIP1 has no effect on Fas-induced apoptosis.^10, 11^ Subsequently, RIP1 was also found to associate with the signaling complex of TNFR1.^12^ It was shown that RIP1 deficiency disrupts NF-*κ*B activation induced by TNFR1 in primary mouse embryonic fibroblast cells or human Jurkat T lymphoma cells.^10, 11^ Several functional domains of RIP1 have been defined. In particular, RIP1 contains a serine/threonine kinase domain (KD) at the amino-terminus and a death domain (DD) at the carboxy-terminus. The intermediate domain, but not the protein serine/threonine KD of RIP1, is required for the activation of NF-*κ*B.^13^ The DD of RIP1 interacts with the DD of TNFR1-associated death domain (TRADD) protein, a signaling adaptor, leading to both apoptosis and NF-*κ*B activation.^12^ Therefore, RIP1 may serve as a scaffold protein in addition to being a protein serine/threonine kinase.

The function of the KD of RIP1 remained unknown until the landmark work by Holler *et al.*,^14^ implicating a novel function for RIP1 in a caspase-independent cell death process with certain characteristics of necrosis, namely necroptosis. Importantly, mutations targeting the kinase activity of RIP1 abolish necroptotic cell death induced by TNFR1. The *in vivo* role of RIP1-mediated necroptosis was first revealed by analysis of the embryonic defect displayed by mice lacking the Fas-associated death domain (FADD) protein.^15^ The FADD adaptor protein relays exclusively apoptotic signals in the pathways triggered by Fas, TNFR1, and TNF-related apoptosis-inducing ligand receptors (TRAIL-Rs or DR4/5).^16, 17, 18^ Whereas none of the DRs are essential for mouse development, FADD deficiency resulted in midgestation death of mouse embryos.^19, 20^ Interestingly, when RIP1 is absent, normal embryonic development is restored in FADD^−/−^ mice,^15^ indicating that FADD^−/−^ embryonic lethality is caused by RIP1-dependent necroptosis.

Although normal during embryogenesis, RIP1^−/−^ FADD^−/−^ double knockout (DKO) mice display perinatal lethality,^15^ similar to the phenotype of RIP1^−/−^ single knockout mice.^10^ In contrast, deletion of a RIP1-related protein kinase, RIP3, fully restores normal embryonic as well as postnatal development in FADD^−/−^ mice.^21^ Recent studies demonstrated that RIP1^−/−^ mice can only reach adulthood when both FADD and RIP3 are absent, indicating that RIP1 protects neonatal cells from FADD-mediated apoptosis and RIP3-dependent necroptosis.^22, 23, 24, 25^ Importantly, FADD^−/−^ RIP3^−/−^ DKO mice and RIP1^−/−^ FADD^−/−^ RIP3^−/−^ triple knockout mice develop age-dependent lymphadenopathy and splenomegaly, reminiscent of the lymphoproliferative (*lpr*) disease displayed by Fas^−/−^ mice. Therefore, both apoptosis and necroptosis are essential for homeostasis in the peripheral lymphoid organs.

Previous studies have indicated that RIP1 is essential for T-cell development, because RIP1-deficient fetal liver cells fail to reconstitute the T-cell compartment in immunodeficient recipient mice.^15, 26^ A recent study showed that lack of RIP1 in hematopoietic stem cells and progenitors (HSCs/Ps) leads to a severe defect in hematopoiesis.^27^ However, the temporal requirement for RIP1, particularly at postlineage commitment stages, remains unclear. In the current study, T lineage-specific deletion of RIP1 revealed a novel stage-specific requirement for RIP1 to protect T cells from apoptosis as well as to allow normal proliferative responses.

## Results

### RIP1 is dispensable in thymic immature T cells but essential for mature T-cell maintenance

**Figure 1:**
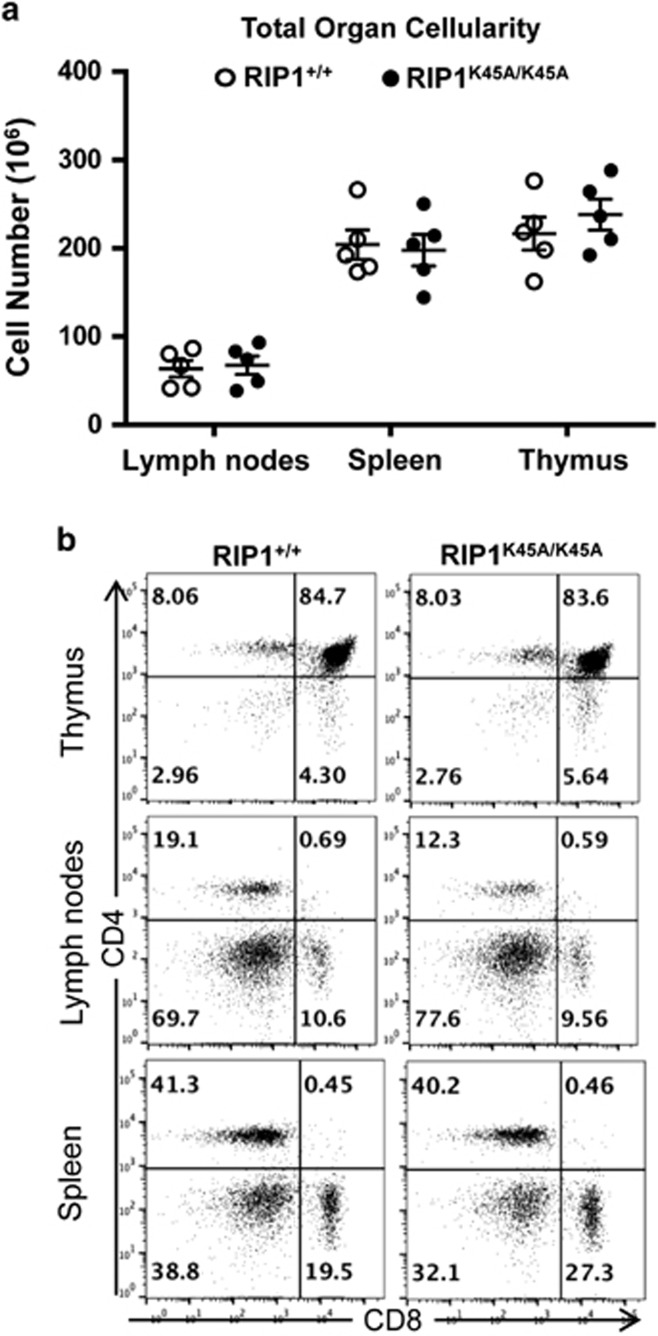
The kinase activity of RIP1 in T cells. (**a**) Cells were isolated from the indicated lymphoid organs of wild-type RIP1^+/+^ mice and mutant mice homozygous for the K45A mutation, and the cells were enumerated. Error bars are average±S.E.M. (*n*=5). *P-*values >0.05. (**b**) T-cell subsets of the indicated lymphoid organs were analyzed by two-color flow cytometry upon staining for the CD4 and CD8 T lineage markers. Representative of at least five independent experiments

In previous studies, deletion of RIP1 in germ cells resulted in perinatal lethality,^10^ and few mature T cells were generated from RIP1^−/−^ fetal liver cells.^15, 26^ More recently, studies found deletion of RIP1 in HSCs/Ps led to lymphocyte deficiency.^27^ However, it remains unclear what role RIP1 has in T cells during postlineage commitment stages. To this end, a mouse line (RIP1^f/f^) carrying a conditional knockout allele of RIP1 was used in the current study, in which exon 3 encoding part of the KD of RIP1 was flanked by two loxP sites.^28^ Moreover, the lysine 45 residue was changed to alanine (K45A) in the floxed exon 3 to disable the kinase activity of RIP1 and thereby interrupt its necroptotic function. The lymphoid compartment of K45A mice was analyzed. Total cellularity of the thymus, spleen, and lymph nodes were comparable between K45A mutant and control RIP1^+/+^ mice (Figure 1a). Flow cytometric analysis showed that the T-cell subsets in the thymus, spleen, and lymph nodes of K45A mutant mice were similar to those in RIP1^+/+^ control mice (Figure 1b). Therefore, the RIP1 conditional allele containing the K45A mutation does not have a detectable effect on T-cell development and maturation.

**Figure 2:**
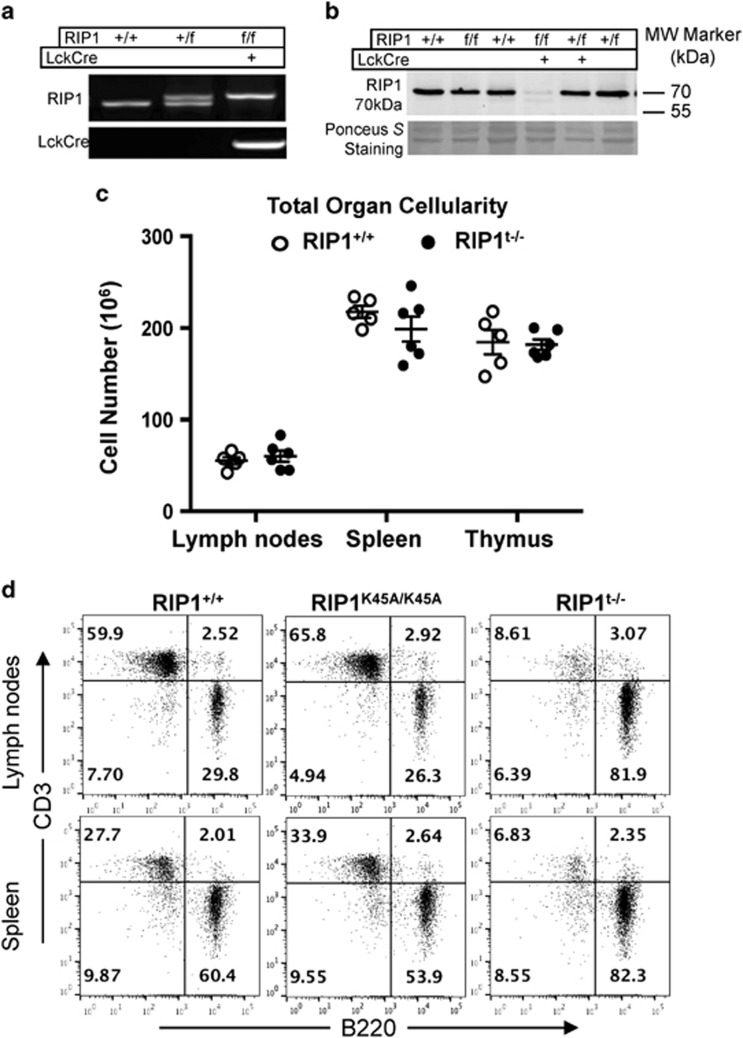
T-cell-specific deletion of RIP1. (**a**) Genotyping by PCR of mouse ear DNA showing genotypes generated from a RIP1^+/f^ × RIP1^+/f^ Lckcre^+^ mating cross. (**b**) Western blotting analysis of RIP1 in thymocytes from mice of the indicated genotypes. Ponceus *S* staining was performed as protein loading/transfer control. (**c**) Total organ cellularity of RIP1^t−/−^ mutant mice (filled circles) and RIP1^+/+^ control mice (open circles) are shown. Error bars are average±S.E.M. *P*-values >0.05. (**d**) Representative two-color flow cytometric plots showing the T cell (CD3^+^) and B cell (B220^+^) populations in the indicated peripheral lymphoid organs

Western blotting analysis was then performed, and it showed that the RIP1^f/f^ mice express the K45A mutant RIP1 protein at levels similar to those of the endogenous RIP1 protein in control RIP1^+/+^ mice (Figures 2a and b). Therefore, the loxP sites and the K45A mutation do not affect the gene expression or protein stability of RIP1. To induce T cell-specific deletion of RIP1, we crossed the LckCre transgene^29^ into the RIP1^f/f^ mice (Figure 2a). LckCre-mediated deletion of floxed alleles is initiated at the pro/pre-T-cell stage, as shown in our previous study.^30^ Western blotting analysis showed that the expression of RIP1 was reduced to minimal levels in the thymus of RIP1^f/f^ LckCre^+^ mutant mice compared with control mice (fourth lane, Figure 2b). This indicates that the LckCre activity leads to efficient deletion of RIP1 in the T lineage in the thymus. Hereafter, RIP1^f/f^ LckCre^+^ is designated RIP1^t−/−^ mice and RIP1^f/f^ mice are the same as RIP1^K45A/K45A^ mice.

**Figure 3:**
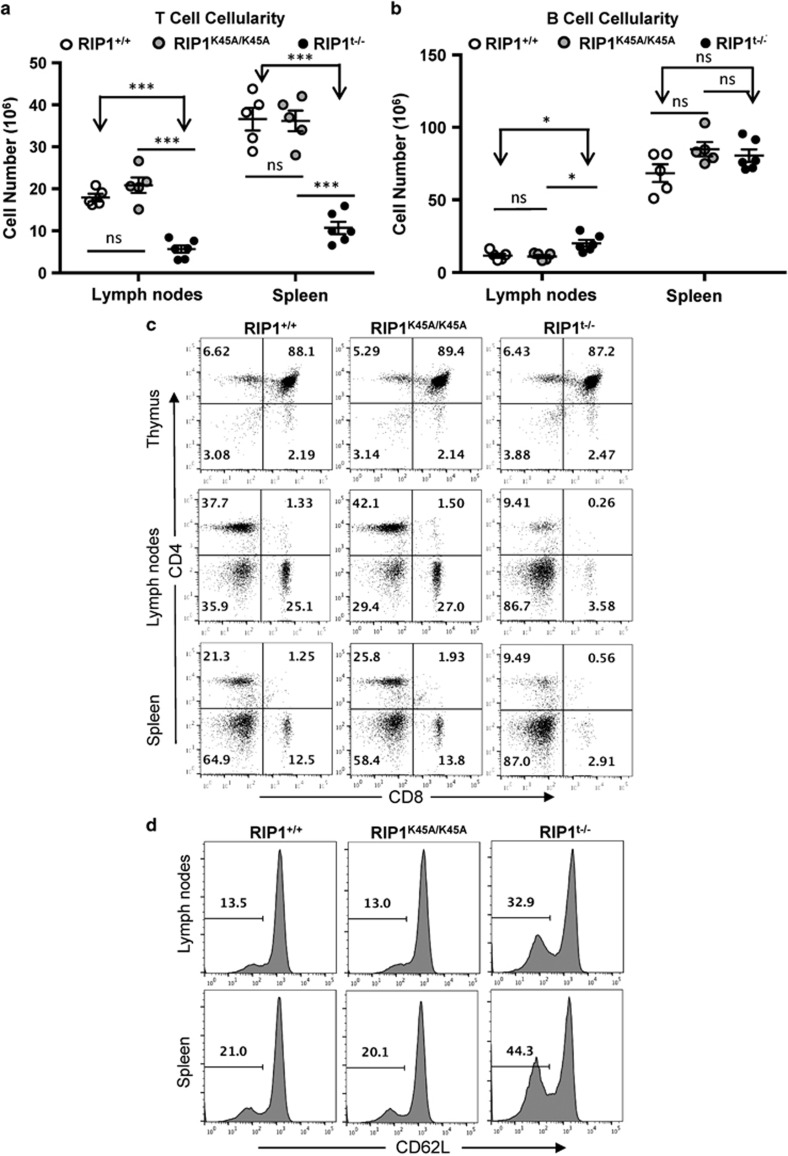
Analysis of T-cell populations in RIP1^t−/−^ mice. (**a**) Absolute T cell (CD3^+^) and (**b**) B cell (B220^+^) numbers in the spleen and lymph nodes of mutant and control mice. *P<0.05, ***P<0.001, NS=not significant. (**c**) T-cell subsets of the indicated lymphoid organs were analyzed by flow cytometry upon staining for the CD4 and CD8 T lineage markers or (**d**) CD62L. Data represents at least six independent experiments

The RIP1^t−/−^ mice live to adulthood with no obvious difference from littermate controls by appearance or body weight (data not shown). The total cellularity of the thymus, spleen, and lymph nodes of RIP1^t−/−^ mice were not significantly different from that in control mice (Figure 2c), and the organ size was normal by appearance and weight (Supplementary Figure S1). Flow cytometric analysis was then performed upon staining cells of the primary and peripheral lymphoid organs for CD3 and B220. The percentages of T-cell populations (CD3^+^) in the periphery (the spleen and lymph nodes) were dramatically decreased in RIP1^t−/−^ mutant mice, in comparison with control littermates (Figure 2d). As a result, the absolute T-cell numbers in the mutant spleen (10.7 × 10^6^±1.5) and lymph nodes (5.6 × 10^6^±0.9) were dramatically lower than RIP1^+/+^ T-cell numbers in the spleen (36.6 × 10^6^±2.7) and lymph node (17.9 × 10^6^±0.8) and RIP1^K45A/K45A^ T-cell numbers in the spleen (36.2 × 10^6^±2.5) and lymph node (20.9 × 10^6^±1.8) (Figure 3a). This T lymphopenic condition resulted in a compensatory expansion and significant increase of B cells in the RIP1^t−/−^ lymph node (Figure 3b) while the myeloid populations of these organs remained unchanged (Supplementary Figure S2). In total, RIP1 deletion leads to more than three-fold reduction of mature T cells in the periphery.

Further flow cytometric analysis of the T lineages was performed, and it showed that the percentages of pro/pre-T cells (CD4^−^CD8^−^), immature T cells (CD4^+^CD8^+^), and mature CD4^+^ and CD8^+^ T cells were similar between RIP1^t−/−^ mutant mice and RIP1^+/+^ and RIP1^K45A/K45A^ control mice (Figure 3c). However, dramatic reductions in the percentages of both CD4^+^ and CD8^+^ lineages of mature T cells were observed in peripheral lymph tissues of RIP1^t−/−^ mice, including the spleen and lymph nodes (Figure 3c). Interestingly, a higher percentage of T cells in RIP1^t−/−^ mice displayed lower expression of CD62L compared with control mice (Figure 3d).

### RIP1 is required for proliferative responses in mature T cells

**Figure 4:**
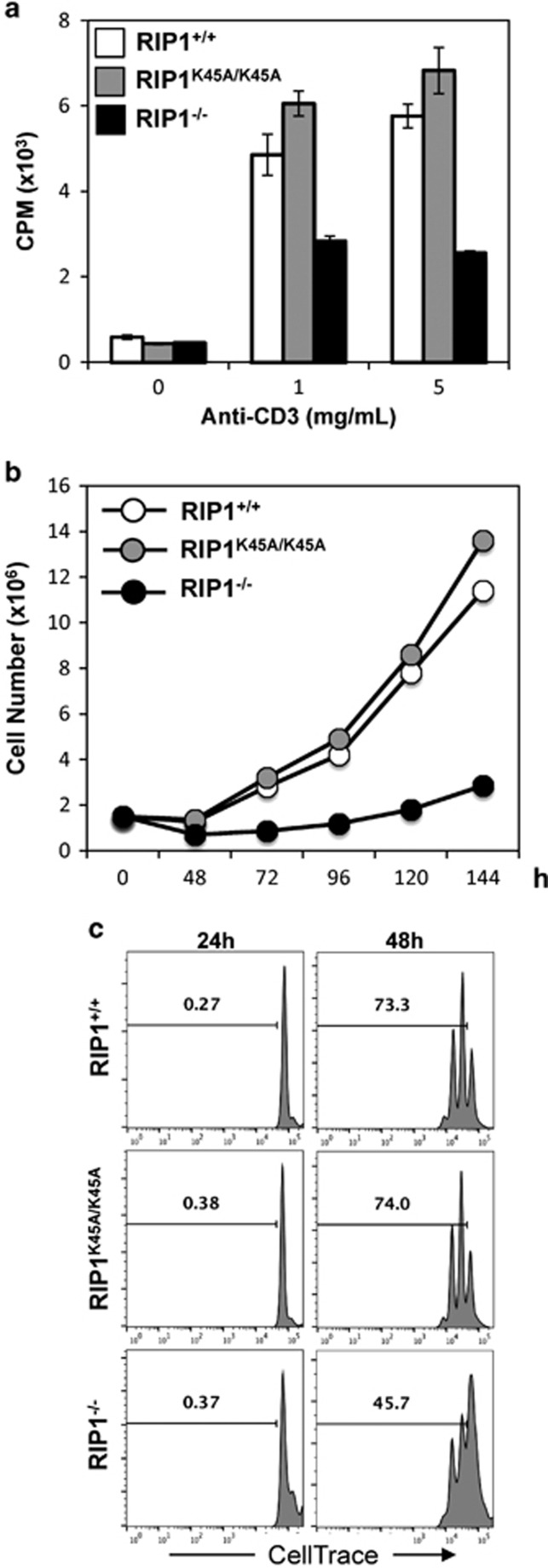
Analysis of cell proliferation responses of activated RIP1^−/−^ T cells. (**a**) Mature T cells were stimulated with the indicated concentrations of anti-CD3 plus anti-CD28 (0.2 *μ*g/ml) Abs for 48 h, and proliferation was determined by [^3^H] thymidine incorporation. Error bars represent±S.D. of triplicate. cpm, count per minute. (**b**) T-cell numbers following activation with anti-CD3 (1 *μ*g/ml)/CD28 (0.2 *μ*g/ml) Abs were enumerated at the indicated times and growth curves were plotted. (**c**) Mature T cells were labeled with CellTrace violet, activated as in panel (**b**), and flow cytometric analysis was performed. The gates indicate divided T cells and the first peak to the right represents undivided T cells at each indicated time point. Histograms are showing live T cells. Numbers indicate the percentages of divided T cells at each time point. Data are representative of three independent experiments

Stimulation through the T-cell antigen receptor (TCR) leads to proliferative responses in mature T cells. To determine whether RIP1 has a role in these proliferative responses, mature T cells were isolated and purified from the lymph nodes and spleen. Stimulation of mature T cells was initiated by crosslinking the TCR with agonistic anti-CD3 antibodies, and co-stimulation was provided by crosslinking CD28 with the corresponding antibodies. T-cell proliferation was measured by the amounts of tritiated thymidine incorporated through DNA synthesis. When compared with RIP1^+/+^ and RIP1^K45A/K45A^ control T cells, a dramatic defect in proliferation responses was observed in RIP1^t−/−^ T cells (Figure 4a). In further analyses of proliferative responses, increases in T-cell numbers were determined at various time points after activation and growth curves were generated. The numbers of activated RIP1^−/−^ T cells barely increased during a 6-day period in culture, whereas exponential growth ensued in activated RIP1^+/+^ and RIP1^K45A/K45A^ T cells (Figure 4b).

Given the severely impaired proliferative responses in RIP1^−/−^ T cells, we analyzed T-cell division kinetics. Mature T cells were purified from peripheral lymphoid organs, prelabeled with CellTrace violet, and then stimulated with anti-CD3 and -CD28 antibodies. When T cells divide, the CellTrace dye is diluted, resulting in lower fluorescence in daughter cells. At various times after stimulation of the TCR, T cells were analyzed by flow cytometry. Cell division was evident in RIP1^+/+^ control T cells (73.3%, top right panel, Figure 4c) and RIP1^K45A/K45A^ T cells (74%, middle right panel, Figure 4c) at 48 h after stimulation, as indicated by the population with lower CellTrace fluorescence. Minimal cell division occurred in RIP1^+/+^, RIP1^K45A/K45A^, or RIP1^−/−^ mutant T cells at 24 h after stimulation. At 48 h, cell division was significant in RIP1^−/−^ T cells (45.7%, lower right panel, Figure 4c). However, this division was much less than that in RIP1^+/+^ and RIP1^K45A/K45A^ T cells. These results indicate that normal proliferative responses in mature T cells require RIP1.

### Death receptor-induced cell death responses in RIP1^−/−^ T cells

**Figure 5:**
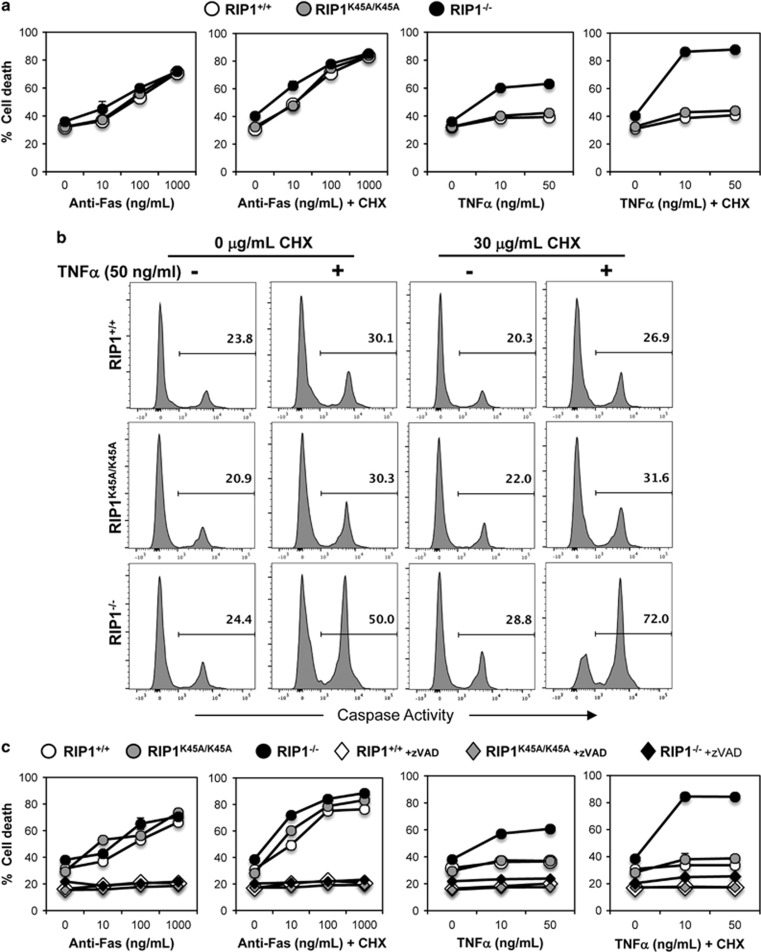
Death receptor responses in RIP1^−/−^ T cells. Thymocytes were treated as indicated with or without 30 *μ*g/ml CHX for 16 h. (**a**) Thymocytes were stained with PI, and the percentages (mean±S.D. of triplicate) of cell death (PI^+^) were determined by flow cytometry. (**b**) CellEvent Caspase 3/7 Green Detection Reagent was incubated with thymocytes for 30 min after treatment; representative flow plots show caspase activity via cleavage of the fluorogenic caspase substrate. (**c**) Thymocytes incubated and analyzed as in panel (**a**) with the addition of 50 *μ*M zVAD where indicated. Data are representative of three independent experiments

Given the defects in mature T-cell maintenance and proliferative responses in RIP1^t−/−^ mice (Figures 1-4), we sought to determine whether lack of RIP1 impacts death responses in RIP1^−/−^ T cells. RIP1^+/+^, RIP1^K45A/K45A^, and RIP1^−/−^ thymocytes readily underwent Fas-induced apoptosis when stimulated with an agonistic anti-Fas antibody and was more pronounced with the addition of cycloheximide (CHX) (left, Figure 5a). However, RIP1^+/+^ wild-type and RIP1^K45A/K45A^ mutant thymic T cells were resistant to TNF treatment. Interestingly, RIP1^−/−^ T cells displayed hypersensitivity to TNF-induced death responses and became more sensitive with the addition of CHX (right, Figure 5a). To investigate the mechanistic nature of the observed cell death induced by TNF*α* in RIP1^−/−^ T cells, we analyzed caspase activities by intracellular staining with cell-permeable fluorogenic caspase substrates.^31^ As shown in Figure 5b (left), untreated RIP1^−/−^ mutant thymocytes contain basal levels of caspase activities (24.4%) that are similar to that in untreated RIP1^+/+^ control (23.8%) and RIP1^K45A/K45A^ (20.9%) T cells. In contrast, greatly higher caspase activities were detected in RIP1^−/−^ T cells treated with TNF*α* and CHX (72%), than in RIP1^+/+^ (26.9%) and RIP1^K45A/K45A^ (31.6%) T cells (right, Figure 5b). To analyze this observation further, we used the pan-caspase inhibitor zVAD, which effectively blocked apoptosis induced by crosslinking of Fas with the agonistic antibody (left, Figure 5c). Moreover, zVAD effectively prevented death in RIP1^+/+^, RIP1^K45A/K45A^, and RIP1^−/−^ thymocytes treated with TNF*α* (right, Figure 5c). This finding indicates that RIP1 inhibits caspase-dependent apoptosis induced by TNF*α* in thymocytes. However, RIP1 does not protect against Fas-induced apoptosis in thymocytes.

### RIP1 inhibits apoptosis during TCR-induced proliferative responses

**Figure 6:**
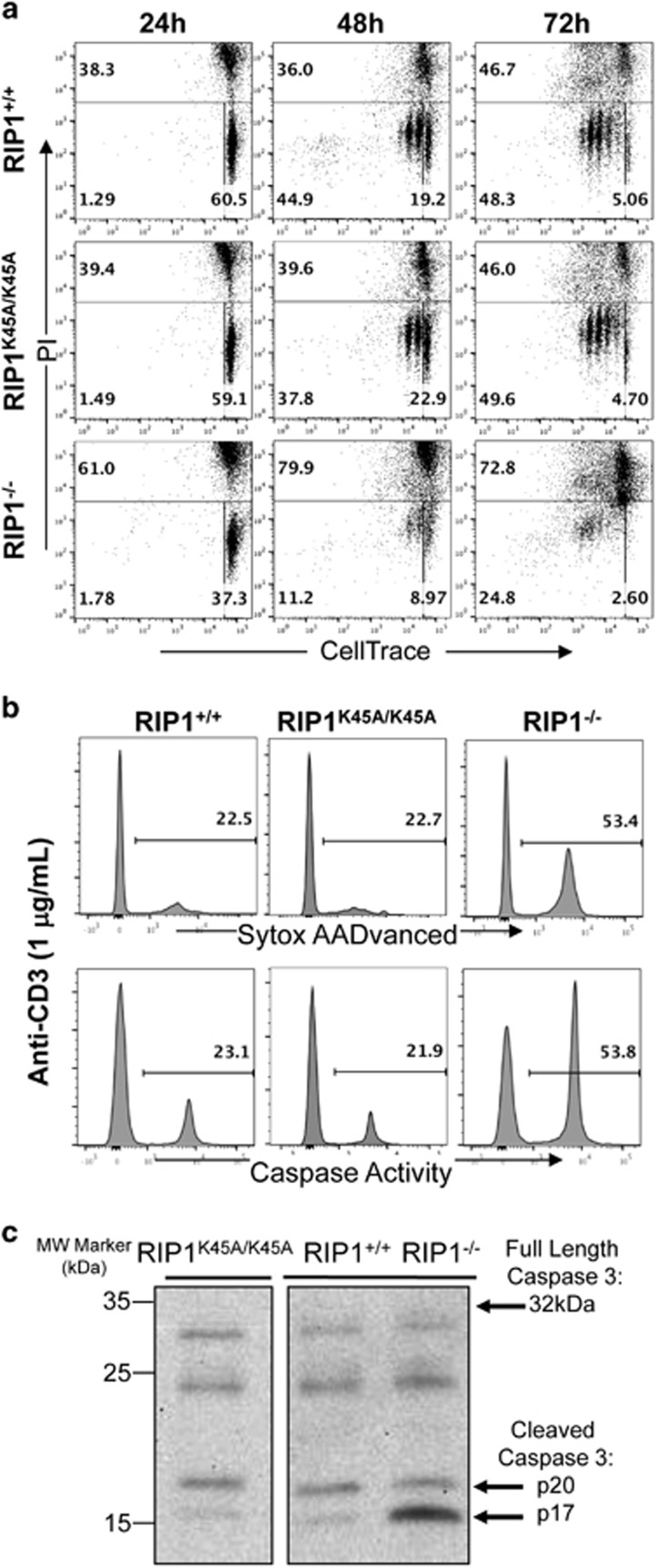
Cell death responses during TCR-induced activation. (**a**) Mature T cells were isolated from the periphery, labeled with Celltrace Violet, and stimulated with anti-CD3 (1 *μ*g/ml) and anti-CD28 (0.2 *μ*g/ml) antibodies for the indicated times. Mature T cells were harvested, stained with PI, and analyzed by two-color flow cytometry. (**b**) Mature T cells were isolated and stimulated as in panel (**a**) for 16 h. T cells were then incubated with CellEvent Caspase 3/7 Green Detection Reagent for 30 min or stained with SYTOX AADvanced Dead Cell Stain and analyzed using a flow cytometer. (**c**) Western blotting of peripheral T cells with the indicated genotype for caspase 3 after stimulation with anti-CD3/CD28 for 48 h. Data are representative of three independent experiments

TCR-induced proliferative responses are accompanied by cell death in wild-type mature T cells. It is unclear whether the observed proliferation defect in RIP1^−/−^ T cells (Figure 4) is due to increased cell death responses. To test this possibility, we isolated mature T cells from the peripheral lymphoid organs and stimulated them through the TCR and CD28. At various times after activation, cell death was analyzed by staining with propidium iodide (PI) and flow cytometry. Significant cell death (35–50% PI^+^) in the activated wild-type T cells was observed at every time point analyzed (top, Figure 6a). However, compared with the RIP1^+/+^ and RIP1^K45A/K45A^ control T cells, greatly increased death was detected in RIP1^−/−^ mutant T cells, as indicated by higher PI^+^ populations (60–80% PI^+^) at all three time points (bottom *versus* top and middle panels, Figure 6a).

In the same assay, T cells were prelabeled with CellTrace. Two-color flow cytometry allows simultaneous analyses of T-cell proliferative responses. At 24 h after stimulation, RIP1^+/+^, RIP1^K45A/K45A^, and RIP1^−/−^ T cells did not divide (left column, Figure 6a). At 48 h after stimulation, 44.9% of control RIP1^+/+^ T cells and 37.8% of RIP1^K45A/K45A^ T cells had divided one or more times (middle column, Figure 6a). However, there was minimal division of RIP1^−/−^ T cells at this time point (11.2%, middle, Figure 6a). Similarly, at a later time point (72 h), there was dramatically less cell division in RIP1^−/−^ T cells (24.8%) than in control RIP1^+/+^ T cells (48.3%) or RIP1^K45A/K45A^ T cells (49.6%). These data indicate that RIP1 deletion resulted in defective T-cell division as well as enhanced cell death.

Further analysis was performed to determine whether enhanced cell death in activated RIP1^−/−^ T cells was the result of apoptosis, which is dependent on caspase activation. To do so, mature T cells were activated through stimulation of CD3 and CD28 and labeled by cell-permeable substrates, which turn fluorogenic upon caspase cleavage.^31^ Intracellular caspase activity was then measured by flow cytometry. Interestingly, activated RIP1^−/−^ T cells contain greatly increased caspase activity compared with RIP1^+/+^ and RIP1^K45A/K45A^ T cells that had been activated for 16 h (Figure 6b). This caspase activity also correlated with an increase in cell death, as shown by positive staining with SYTOX AADvanced dead cell stain at the 16 h time point (Figure 6b). Furthermore, western blotting analysis also showed increased cleavage of pro-caspase 3 in activated RIP1^−/−^ T cells (Figure 6c). These results indicate that RIP1 promotes survival by suppressing TCR-induced caspase activation.

**Figure 7:**
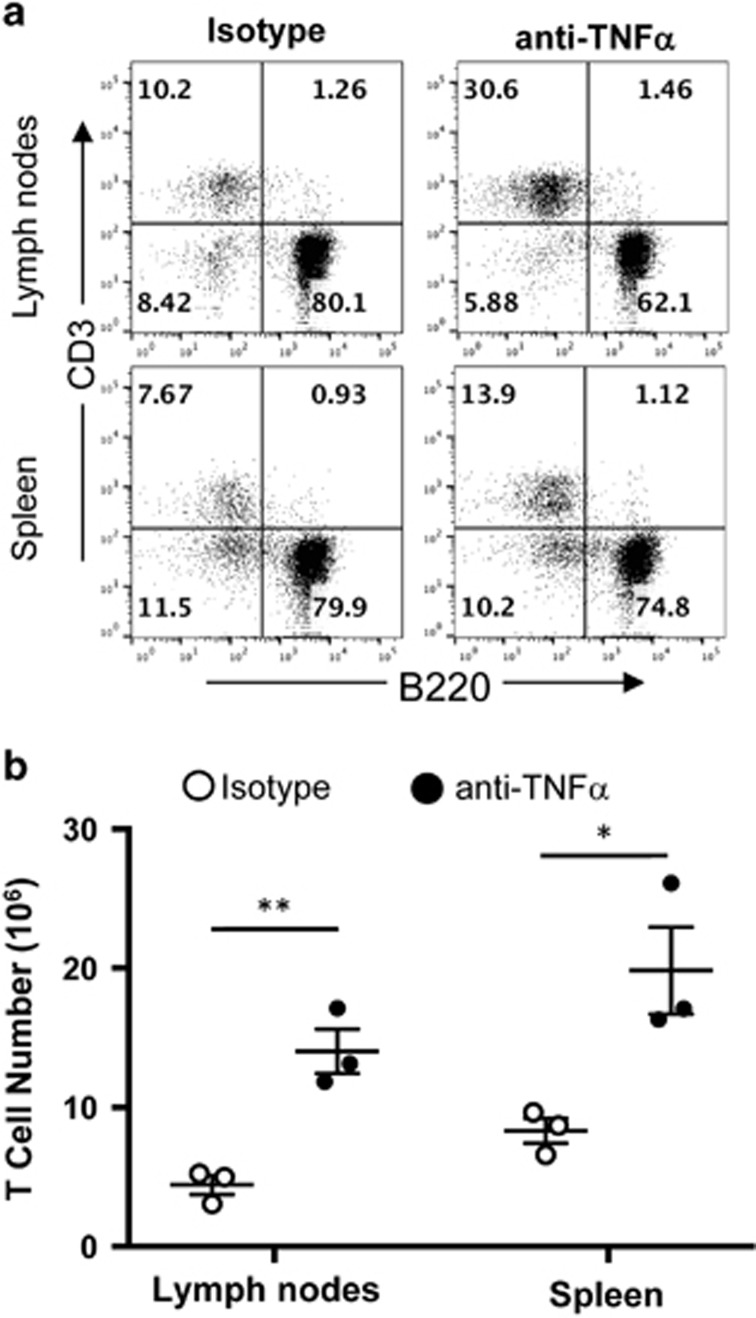
*In vivo* TNF*α* blockade in RIP1^t−/−^ mice. (**a**) Representative two-color flow cytometric plots showing the T cell (CD3^+^) and B cell (B220^+^) and (**b**) total T-cell numbers in the indicated peripheral lymphoid organs of RIP1^t−/−^ mice treated with anti-TNF*α* blocking antibody or isotype control for 2 weeks. *P<0.05, **P<0.01

Given that RIP1 is protecting T cells from increased caspase activity and apoptosis, we next investigated a potential *in vivo* trigger that could cause the RIP1^t−/−^ peripheral T-cell defect. As RIP1^−/−^ thymocytes are sensitive to TNF*α*-mediated apoptosis *in vitro*, an *in vivo* TNF*α* blockade was performed by treating RIP1^t−/−^ mice with anti-TNFa blocking antibody or isotype control every 3.5 days. After 2 weeks, the percentage of CD3^+^ T cells in the spleen and lymph nodes had increased (Figure 7a) and resulted in a significant rescue of peripheral T-cell numbers in the spleen and lymph nodes (Figure 7b). This indicates that RIP1 helps maintain T-cell homeostasis by protecting T cells from TNF*α*-mediated apoptosis.

## Discussion

Since its initial cloning >20 years ago,^9^ the physiological function of RIP1 in T cells has not been fully understood, mainly owing to perinatal lethality of RIP1^−/−^ mice.^10^ Although RIP1 associates with the signaling complex of TNFR1 or Fas, neither of the two death receptors was essential during mouse development or lymphocyte development. Prior studies have indicated that RIP1 may be essential for T-cell development, because RIP1^−/−^ fetal liver cells failed to reconstitute the T-cell compartment of immune-deficient recipient mice,^15, 26^ and mice lacking RIP1 in HSCs/Ps contain few T cells.^27^ However, our current data showed that RIP1 is dispensable in thymic development, as deletion of RIP1 at the pro/pre-T-cell stage using LckCre resulted in no defect in immature thymocyte profiles (Figures 2 and 3). Although dramatically decreased, mature T cells were present in significant numbers in RIP1^t−/−^ mice (Figures 2 and 3), unlike the near complete blockage of T-cell development when RIP1 is deleted in HSCs/Ps.^26, 27^ These results clearly demonstrate a temporal requirement for RIP1 in HSCs/Ps^26, 27^ and mature T cells (Figures 2 and 3) but less so in pro/pre-T cells or immature T cells (Figure 3). The conditional mouse model also has a selective defect in necroptosis, owing to the K45A mutation inactivating the kinase activity. However, our data show that the kinase activity of RIP1 is dispensable in the T-cell compartment (Figure 1). As the KD of RIP1 has been implicated in necroptosis and not in apoptosis, this further supports the hypothesis that RIP1 is functioning to protect T cells from apoptosis.

Although RIP1 may bind to Fas, cell death responses were largely intact (Figure 5a), indicating that RIP1 is dispensable in Fas-induced signaling. There have been data showing that RIP1 could either promote or inhibit apoptosis induced by TNF*α*. Wild-type primary cells are generally resistant to TNF*α* treatment. We found that deletion of RIP1 dramatically sensitized immature T cells to TNF-induced death responses (Figure 5a). In contrast, intrinsic cell death responses were not affected by a lack of RIP1 in T cells (data not shown). Therefore, RIP1 provides protection against cell death in a pathway-specific manner. Previous studies, including ours, indicate that RIP1 perinatal lethality is due to uncontrolled FADD/caspase 8-mediated apoptosis and RIP3-mediated necrosis. However, T cell-specific ablation of RIP1 demonstrates that its main function in T cells is to primarily protect against apoptosis (Figure 6), not necrosis. This indicates that, while RIP1 does regulate apoptosis and necrosis, it may do so in a cell-type-specific manner, for example, protecting against apoptosis in T cells but protecting against RIP3-mediated necrosis in HSCs/Ps.^27^

We have previously shown that the few T cells derived from adoptively transferred RIP1^−/−^ fetal liver cells displayed a severe defect in proliferation responses upon stimulation of the TCR. However, it was not clear whether this defect was due to abnormal development when RIP1 was absent in HSCs/Ps. This T-cell proliferation defect was recapitulated in the current study, in which RIP1 was deleted in lineage-committed T cells, definitively showing that RIP1 is involved in mature T-cell proliferation (Figures 4a–c). Most importantly, this phenotype may be due not only to increased death in activated T cells but also to impaired cell division capability (Figure 6a). In addition to inhibiting apoptosis and necroptosis, RIP1 has a cell-death-independent function of activating the canonical NF-*κ*B pathway, which has been best studied in the context of TNFR1 signaling. As stimulation of the TCR can activate the NF-*κ*B pathway and the MAPK and AKT pathways, it is possible that TCR-induced activation of the NF-*κ*B pro-survival pathway involves a function of RIP1.

Dysregulation of not only TCR signaling but also uncontrolled cell death can have far-reaching implications for disease progression and proper immune system function. Hyperactivity of the TNF pathway has been associated with several chronic autoimmune and inflammatory diseases, such as psoriasis, rheumatoid arthritis, and multiple sclerosis. Although anti-TNF*α* treatment has been shown to be effective in some cases, a more specific approach, such as targeting RIP1, could have more success and fewer side effects. Uncovering how RIP1 functions, especially in T cells, which are major mediators of autoimmune/inflammatory conditions, is essential to the discovery of targeted therapies and treatments. Alternatively, as this study shows that RIP1 is essential for T-cell maintenance, targeting RIP1 in T cells could be used as a treatment for T-cell leukemia and lymphomas to induce apoptosis and reduce T-cell numbers.

In summary, our data provide clear evidence that RIP1 has a critical role in maintenance of the mature T-cell compartment, which could contribute to immune disorders, including immunodeficiency. However, the kinase activity itself is dispensable in T-cell development and maintenance. Previous studies show that lack of RIP1 causes necroptosis, leading to inflammation in neonatal tissues.^24^ In the adult skin, it appears that the K45A mutation helps suppress inflammatory diseases in mice deficient in SHARPIN.^28^ Therefore, rather than targeting the whole RIP1 protein, selectively blocking the kinase activity, which mediates necroptosis, could be considered as a strategy to help manage certain inflammatory conditions owing to necroptosis.^28^ However, current studies remain incomplete in understanding the complicated regulatory circuitry mediated by RIP1. Further investigations would help reveal more details in the molecular mechanism involving RIP1 and its role in a variety of autoimmune/inflammatory diseases and cancer.

## Materials and Methods

### Mice

Mice containing a conditional knockout allele of RIP1 (RIP1^f/f^ or RIP1^K45A/K45A^) were described elsewhere.^28^ To generate T-cell-specific RIP1 knockout mice, RIP1^f/f^ mice were crossed with LckCre transgenic mice generated in Dr. C Wilson's laboratory^29^ and originally obtained from Taconic (Hudson, NY, USA). All of the animal studies were approved by the Institutional Animal Care and Use committee at Thomas Jefferson University. The LckCre transgene was genotyped by PCR using the primers 5′-CCGAAATTGCCAGGATCAGG-3′ and 5′-CTTACCTGTAGCCATTGCAGCTAG-3′. PCR-mediated genotyping was performed as described. RIP1^+/+^ littermates from RIP1^+/f^ Lckcre^+^ × RIP1^+/f^ mating crosses were used as controls whenever possible. Otherwise, age/sex-matched RIP1^+/+^ mice resulting from RIP1^+/f^ Lckcre^+^ × RIP1^+/f^ mating crosses were used as controls.

### Western blotting analysis

Total thymocytes were isolated from mice with various genotypes. Mature T cells were purified by sorting from the spleen and lymph nodes using a FACS Aria (BD Biosciences, San Jose, CA, USA) and activated by 1 *μ*g/ml anti-CD3 and 0.2 *μ*g/ml anti-CD28 antibodies. Cells were lysed in cold RIPA buffer containing 50 mM Tris pH 8.0, 150 mM NaCl, 1% Nonidet P-40, 0.5% Na-deoxycholate, 0.1% SDS, 1 mM phenylmethyl sulphonyl fluoride, and proteinase inhibitor cocktail (Roche, Indianapolis, IN, USA). Proteins (40 *μ*g) were separated on a 10% or 15% SDS-PAGE gel and transferred to a nitrocellulose membrane. Membranes were stained with Ponceus *S* (Sigma-Aldrich, St. Louis, MO, USA) as a loading/transfer control. Antibodies specific for RIP1 (BD Biosciences, Catalog no. 610459) or caspase 3 (BD Biosciences Catalog no. 611048) were incubated with the membrane overnight at 4 °C then with HRP-conjugated goat anti-mouse antibody (1/10 000) (Vector Laboratories, Burlingame, CA, USA). Chemiluminescent signals were detected using a Signals A FluorChem M Western Blot Imaging machine (ProteinSimple, Inc., San Jose, CA, USA).

### Flow cytometry

Lymph nodes, spleen, and thymus were isolated from 2- to 4-month-old mice of the indicated genotype. Single-cell suspensions were made and red blood cells were depleted via hypotonic lysis. Cells were stained on ice with anti-CD3 and anti-CD4 (BD Pharmingen, San Diego, CA, USA) and anti-CD8 and anti-B220 (Caltag, Buckingham, UK) fluorochrome-conjugated antibodies in PBS containing 3% BSA, 0.5 mM EDTA, and 0.1% sodium azide for 30 min, washed twice with PBS, acquired on an LSR II flow cytometric analyzer (BD Biosciences), and analyzed using the Flowjo software (Treestar, Ashland, OR, USA). Cell numbers were calculated using either a hemacytometer or Countess Automated Cell Counter (Invitrogen, Carlsbad, CA, USA). T-cell numbers were calculated by multiplying total cell number by the percentage of CD3^+^ cells. Unpaired two-tailed *t*-tests were performed to obtain *P*-values comparing the cellularity of RIP1^+/+^, RIP1^K45A/K45A^, and RIP1^t−/−^ mice.

### T-cell proliferation assays

Mature T cells were purified from the spleen and lymph nodes from mice of the indicated genotypes by staining with anti-Thy1.2 antibody (BD Pharmingen) and sorting using a FACS Aria (BD Biosciences). Purified T cells (10^5^) were seeded into 96-well round-bottom plates coated with 1 *μ*g/ml anti-CD3 antibodies in complete RPMI media (cRPMI) that contains 10% FBS, 2 mM L-Glutamine, penicillin (100 U/ml), streptomycin (100 *μ*g/ml), and *β*-mercaptoethanol (5 *μ*M) with the addition of anti-CD28 antibodies (0.2 *μ*g/ml). After 40-h incubation at 37 °C with 5% CO_2_, 1 *μ*Ci of [^3^H] thymindine was added before an additional 8-h incubation. Cells were collected using a Mach Harvester 96 (Tomtec, Hamden, CT, USA) and incorporation was measured using a Wallac 1205 Betaplate Counter (Perkin Elmer, Waltham, MA, USA). For growth curve analysis, purified T cells were seeded in a 24-well plate coated with 1 *μ*g/ml anti-CD3 antibodies in cRPMI with 0.2 *μ*g/ml anti-CD28 antibodies. Cells were enumerated at the indicated times using a Countess Automated Cell Counter (Invitrogen). For cell division kinetics, cells were labeled using the CellTrace Violet Cell Proliferation Kit (Invitrogen), as per the manufacturer's instructions. At the indicated time points, 1 *μ*g/ml PI was added and the cells were analyzed by two-color flow cytometry.

### Cell death assays

Thymocytes (10^5^) were seeded in 96-well flat-bottom plates in cRPMI with various concentrations of either Jo2 monoclonal anti-mouse Fas antibody (BD Pharmingen) or TNF*α* for 16 h. To inhibit caspases, thymocytes were incubated with 50 *μ*M zVAD-fmk for 1 h prior to stimulations. After incubation, 1 *μ*g/ml PI was added and the percentage of cell killing was measured by flow cytometry.

### Caspase activity measurement

CellEvent Caspase 3/7 Green Flow Cytometry Assay Kit (Invitrogen) was used to measure caspase acitivty in thymocytes and mature peripheral T cells as per the manufacturer's instructions. Briefly, after incubation of thymocytes with 50 ng/ml TNF*α* or mature T cells with 1 *μ*g/ml anti-CD3 and 0.2 *μ*g/ml anti-CD28 antibodies for 16 h, CellEvent Caspase 3/7 Green Detection Reagent was added and incubated with cells for 30 min at 37 °C with 5% CO_2_. Samples were analyzed using an LSR II flow cytometric analyzer (BD Biosciences) and analyzed using the Flowjo software (Treestar). In some cases, SYTOX AADvanced Dead Cell Stain (Invitrogen) was used to stain for dead cells. For *in vivo* TNF*α* blockade, mice were treated every 3.5 days for 2 weeks by intraperitoneal injection with 100 *μ*g of anti-TNF*α* antibody (MP6-XT22, Biolegend, San Diego, CA, USA), IgG1 isotype control (Biolegend). After 2 weeks of treatment, lymph nodes and spleen were isolated, single-cell suspensions were made, and lymphocyte populations were determined by FACS analysis using an LSR II (BD Biosciences).

## Abbreviations

RIP1: receptor-interacting protein 1
TNFR1: tumor necrosis factor receptor 1
KD: kinase domain
DD: death domain
TRADD: tumor necrosis factor receptor-associated death domain
FADD: Fas-associated death domain
HSC/P: hematopoietic stem cell and progenitor
TCR: T-cell antigen receptor
CHX: cycloheximide
