# Supplementary figures and images for: Kinase-independent function of RIP1, critical for mature T-cell survival and proliferation
Source: Cell Death Dis. 2016 Sep 29;7(9):e2379–. doi: 10.1038/cddis.2016.307 (PMC5059890; doi:10.1038/cddis.2016.307)

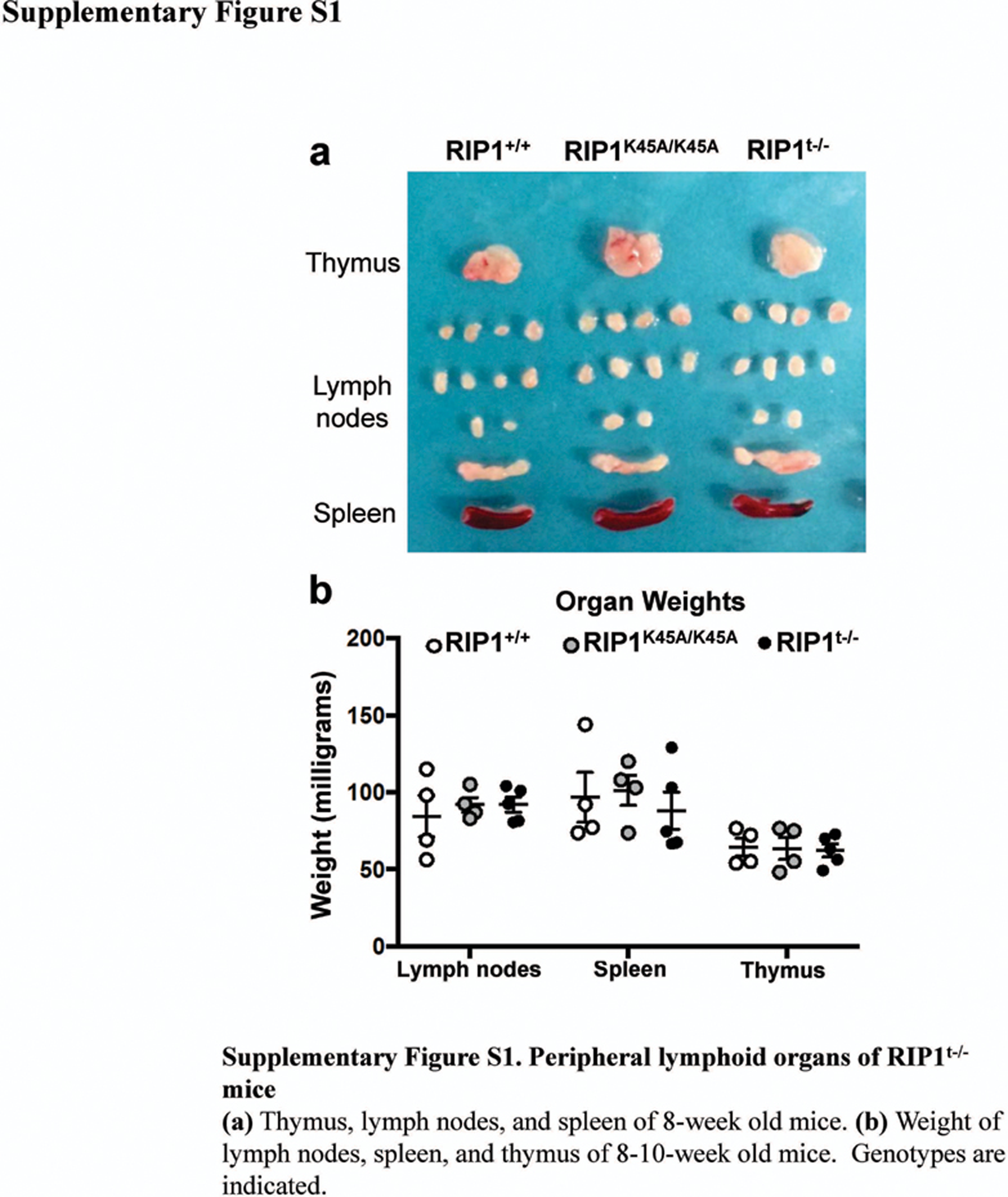

Supplement: Supplementary Figure S1 [file cddis2016307x1.tif]

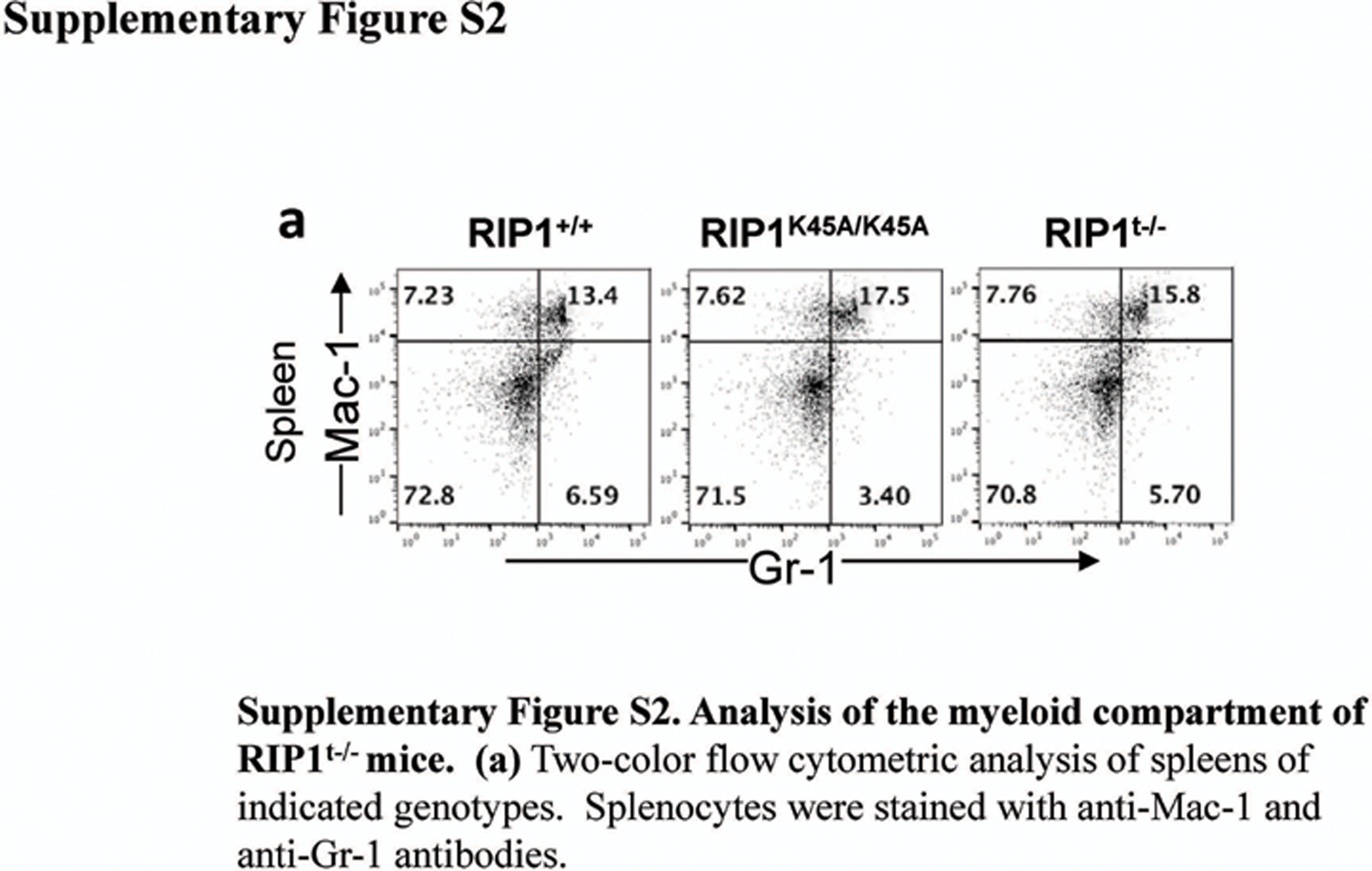

Supplement: Supplementary Figure S2 [file cddis2016307x2.tif]
